# Optimized Atomic Partial Charges and Radii Defined by Radical Voronoi Tessellation of Bulk Phase Simulations

**DOI:** 10.3390/molecules26071875

**Published:** 2021-03-26

**Authors:** Martin Brehm, Martin Thomas

**Affiliations:** Institut für Chemie, Martin-Luther-Universität Halle–Wittenberg, von-Danckelmann-Platz 4, D-06120 Halle (Saale), Germany; martin.thomas@unitybox.de

**Keywords:** atomic partial charges, Voronoi tessellation, molecular dynamics, electron structure theory, numerical integration, non-linear optimization, bulk phase, liquid phase, hydrogen bond

## Abstract

We present a novel method for the computation of well-defined optimized atomic partial charges and radii from the total electron density. Our method is based on a two-step radical Voronoi tessellation of the (possibly periodic) system and subsequent integration of the total electron density within each Voronoi cell. First, the total electron density is partitioned into the contributions of each molecule, and subsequently the electron density within each molecule is assigned to the individual atoms using a second set of atomic radii for the radical Voronoi tessellation. The radii are optimized on-the-fly to minimize the fluctuation (variance) of molecular and atomic charges. Therefore, our method is completely free of empirical parameters. As a by-product, two sets of optimized atomic radii are produced in each run, which take into account many specific properties of the system investigated. The application of an on-the-fly interpolation scheme reduces discretization noise in the Voronoi integration. The approach is particularly well suited for the calculation of partial charges in periodic bulk phase systems. We apply the method to five exemplary liquid phase simulations and show how the optimized charges can help to understand the interactions in the systems. Well-known effects such as reduced ion charges below unity in ionic liquid systems are correctly predicted without any tuning, empiricism, or rescaling. We show that the basis set dependence of our method is very small. Only the total electron density is evaluated, and thus, the approach can be combined with any electronic structure method that provides volumetric total electron densities—it is not limited to Hartree–Fock or density functional theory (DFT). We have implemented the method into our open-source software tool TRAVIS.

## 1. Introduction

The determination of atomic partial charges in chemical systems is important for many fields of computational chemistry, e.g., to parametrize force fields for classical molecular dynamics simulations, which are known to depend sensitively on the atomic charges [1,2,3], or even for estimating the electronic correlation energy [4,5]. As partial charges are not quantum mechanical observables, no canonical way exists to derive them, and a plethora of methods to define these partial charges has been proposed. The most popular techniques can be classified according to a few general ideas. The first approach relies on a partitioning of the molecular wave function expressed in terms of basis functions. This class includes, e.g., Mulliken population analysis [6], Löwdin population analysis [7,8], and natural population analysis (NPA) [9]. Recently, similar tools have become available for plane-wave calculations of solid-state materials [10,11], based on analytical projections of the PAW wave function [12]. Another group of methods is based on the molecular electrostatic potential: a certain set of points is chosen and the partial charges are fitted to reproduce the electrostatic potential given by the molecular wave function at these points [13,14,15]. The commonly applied CHELPG charges [16,17] and RESP charges [18] are, e.g., obtained in this way. The dynamically generated RESP charges [19,20,21] follow a very similar idea. Further techniques are closely related to the multipole moments of the molecular charge distribution, e.g., generalized atomic polar tensor charges [22], density derived atomic point charges (DDAPC, Blöchl charges) [23], and local multipole-derived charges [24].

Atomic partial charges obtained by spatially partitioning the electron density have also been of interest for a long time. There exist two general concepts that use either diffuse weighting functions on the atoms, or distinct boundaries between them. In the former concept introduced by Hirshfeld [25], the weighting functions are derived from a pro-molecular density that consists of the spherically averaged ground-state atomic densities. The definition of the weighting functions was modified later on to extend the applicability of this approach to a broader range of systems [26,27,28,29]. Stewart atoms are also diffuse and they possess a spherical shape that is fitted to reproduce the molecular electron density as close as possible [30,31]. Concerning the second concept, distinct boundaries between the atoms can be obtained by a topological analysis of the electron density as in Bader’s theory of atoms in molecules [32,33]. Another possibility are purely geometric criteria such as the Voronoi tessellation [34,35,36], where a simple plane is placed midway between two atoms [37]. This idea was extended later on to account for different atom sizes by shifting the boundary planes [38,39,40,41], for example in Richards’ “method B” [42] which found some applications [40]. A combination of both general concepts discussed above are Voronoi deformation density charges, where the deformation density of Hirshfeld’s approach is integrated in Voronoi cells around the atoms [43].

In this article, we present a new method to calculate atomic partial charges by partitioning the electron density. We follow the previous idea of using the distinct boundaries of a Voronoi tessellation, but we employ a generalization in terms of the radical Voronoi tessellation (also known as “power diagram” in the two-dimensional case) [44]. In this technique, a radius is assigned to each atom, allowing to model the sizes of the atoms in a chemically reasonable sense. Such radii have also been used in reference [40], but instead of the ratio of the radii, the difference of the squared radii determines the position of the cell face between two atoms here. Thus, in contrast to the aforementioned “method B” [40,42] and similar approaches, the radical Voronoi tessellation does not suffer from the “vertex error” [42,44], i.e., it does not contain holes. When integrating electron density, this is important to keep the total charge of the system constant. As another advantage, the Voronoi sites around which the cells are constructed can be kept on the atoms and do not have to be shifted (as it was done in reference [39]) to obtain a chemically reasonable partitioning. To the best of our knowledge, the radical Voronoi tessellation has not been used for the computation of atomic partial charges before.

The crucial parameters in the radical Voronoi tessellation are the radii assigned to the atoms. We have recently shown [45] that van der Waals radii [46,47,48] yield a reasonable separation of molecules in the bulk phase, and that the resulting molecular electromagnetic moments can readily be used to calculate vibrational spectra of bulk phase systems from ab initio molecular dynamics (AIMD) simulations, including infrared [45,49,50], Raman [45], vibrational circular dichroism (VCD) [51], Raman optical activity (ROA) [52], and resonance Raman [53] spectra. Since van der Waals radii have been fitted to reproduce intermolecular distances, it can be expected that they lead to a suitable placement of the molecular boundaries in a radical Voronoi tessellation. For the assignment of atomic partial charges, however, it is also important to partition the electron density within the molecules in an appropriate way, and another set of radii might perform better in this regard. In the Supporting Information, we show that a single set of radii is not suitable to yield reasonable results for both molecular and atomic charges at the same time. Therefore, we have developed a two-step approach, which first tessellates the total simulation cell into molecular volumes by a first set of radii, and subsequently tessellates each molecular volume into atomic cells by a second set of radii. As a simple criterion to generally assess the quality of a certain set of radii, we propose that the standard deviation in both molecular and atomic charge distributions sampled by an ab initio molecular dynamics simulation should become minimal. With the aid of an optimization algorithm, this leads to a rigorous definition of two sets of atomic radii, and it yields molecular charges as well as atomic partial charges in bulk phase systems which are free of any empirical or adjustable parameters.

At the example of several simple organic compounds in the liquid phase, we will show that the atomic partial charges obtained in this way are chemically reasonable and can help to understand the specific interactions present in the systems, such as hydrogen bonds [54,55]. Furthermore, the application to ionic liquid systems will reveal that the ionic partial charges reflect the charge transfer effects (i.e., reduced ionic charges below unity) that have been discussed for such systems [1,2,3,56,57,58,59]. In many force field molecular dynamics studies of ionic liquids up to now, the ion charges have been empirically scaled down to achieve this effect [1,2,3,57,58,60,61,62], which is required to obtain the correct time scale of the dynamics. However, it has been recently shown that a simple scaling-down procedure of atomic charges might break the subtle balance of interactions within such complex systems [63]. We would like to mention that there exist also other studies which have utilized different methods to obtain reduced ion charges without manual down-scaling [63,64,65,66,67].

The article is structured as follows: in Section 2, we give a general overview of our novel method, followed by some in-depth details of the algorithms. In the next section, the application of the method to several example systems is described and the results are discussed. Subsequently, we show that a simple one-step scheme without optimized radii fails to produce reasonable results (Supporting Information). Finally, we investigate the basis set dependence of our approach. After the computational details, we end the article with a conclusion.

## 2. Description of the Method

### 2.1. General Workflow

As stated in the introduction, the Voronoi tessellation has been used for the calculation of atomic partial charges before. This is also true for several extensions of the Voronoi tessellation which include atomic radii [38,39,40,41]. However, to the best of our knowledge, the radical Voronoi tessellation has not been employed to derive atomic partial charges before. Apart from this, the central piece (and the novelty) of the herein presented approach is the use of two different sets of radii at the same time in our two-step scheme, and the on-the-fly optimization of the atomic radii by minimization of the charge fluctuations. The rationale behind this procedure is the assumption that molecular charges as well as atomic partial charges from a proper partitioning of the electron density should stay approximately constant during the scope of a simulation, and only fluctuate around an average value for a system without significant charge transfer [68,69]. The radical Voronoi tessellation is utilized as a partitioning scheme which requires some parameters to be set (i.e., the atomic radii). Based on the assumption made above, the optimal set of parameters is obtained when the variance of the charge distribution of atoms/molecules becomes minimal. In this case, the tessellation scheme assigns the electron density to the atoms/molecules in a natural way, such that the fluctuations in the charge become small. Therefore, our approach is free of any empirical or adjustable parameter.

The next step is to decide which radii to include into the set of parameters that is optimized for minimal charge fluctuations. One possibility would be to consider only element radii as parameters, and assume all atoms of a certain element type to bear the same radius. However, this would contradict the nature of chemical bonding. It would seem quite counter-intuitive to constrain, e.g., a carboxyl carbon atom to possess the same atomic radius as a methyl carbon atom somewhere else in the molecule. On the other hand, it is also not a good idea to include the radii of all individual atoms into the set of optimization parameters, as atoms which are chemically equivalent should probably bear the same radius. A bulk phase water simulation should require, e.g., only two parameters to optimize, namely the oxygen radius and the hydrogen radius. Based on these considerations, our method groups chemically equivalent (strictly speaking: *topologically equivalent* [70]) atoms together, and uses one atomic radius for each such group as optimization parameter. Similarly, all atoms within one such group are considered to have the same atomic partial charge on average, only influenced by temporal fluctuations.

The objective of the optimization problem is the charge variance. This can either be defined as the average variance of the charge distribution of chemically equivalent atoms (which is then called *atomic charge variance*), or as the average variance of the charge distribution of equivalent molecules (being called *molecular charge variance*). In both cases, the variance is evaluated over the simulation cell (iterating through equivalent atoms or molecules) as well as over the number of snapshots considered. This can be written as

(1) $$\mathrm{Var}:=\frac{1}{K}\sum_{i=1}^{K}\left(\frac{1}{TN_{i}}\sum_{t=1}^{T}\sum_{j=1}^{N_{i}}{\left(q_{i,t,j}-\mu_{i}\right)}^{2}\right)$$

with the average value $\mu_{i}$ given by

(2) $$\mu_{i}:=\frac{1}{TN_{i}}\sum_{t=1}^{T}\sum_{j=1}^{N_{i}}q_{i,t,j},$$

where *T* is the number of snapshots that are to be evaluated. In the case of the atomic charge variance, *K* is the number of non-equivalent atom types in the system, $N_{i}$ depicts how many equivalent atoms of type *i* exist, and $q_{i,t,j}$ is the charge of the *j*-th atom of type *i* in snapshot *t*. In the case of the molecular charge variance, similarly, *K* is the number of non-equivalent molecule types in the system, $N_{i}$ depicts how many molecules of type *i* exist, and $q_{i,t,j}$ is the total charge of the *j*-th molecule of type *i* in snapshot *t*. When presenting the results, the standard deviation σ is used instead of the variance in order to have the numbers in units of charge, simply defined as $\sigma :=\sqrt{\mathrm{Var}}$.

Following from these definitions, a minimization of the atomic charge variance will lead to a tessellation scheme which distributes the electron density to the atoms of the system in a natural way, not considering that there exist molecules at all. On the other hand, the minimization of the molecular charge variance will yield a partitioning which accurately describes the distribution of electron density to different molecules, but does not account at all for the way how the density is distributed to the atoms within each molecule. Since average atomic point charges as well as average molecular/ionic charges are important quantities, both optimization problems are interesting for sure. However, it would be desirable to obtain a method which addresses both problems simultaneously, giving reasonable atomic point charges as well as meaningful molecular charges in one pass. In order to minimize both the molecular and the atomic charge variance, we have developed a two-step approach. We first perform a radical Voronoi tessellation based on a first set of atomic radii $\left\{r^{\mathrm{Mol}}\right\}$ to partition the total simulation cell into molecular volumes. For each such molecular volume, we perform a second radical Voronoi tessellation with a second set of atomic radii $\left\{r^{\mathrm{Atom}}\right\}$ in order to obtain the atomic Voronoi cells. Both sets of radii are optimized independently during the procedure.

**Figure 1:**
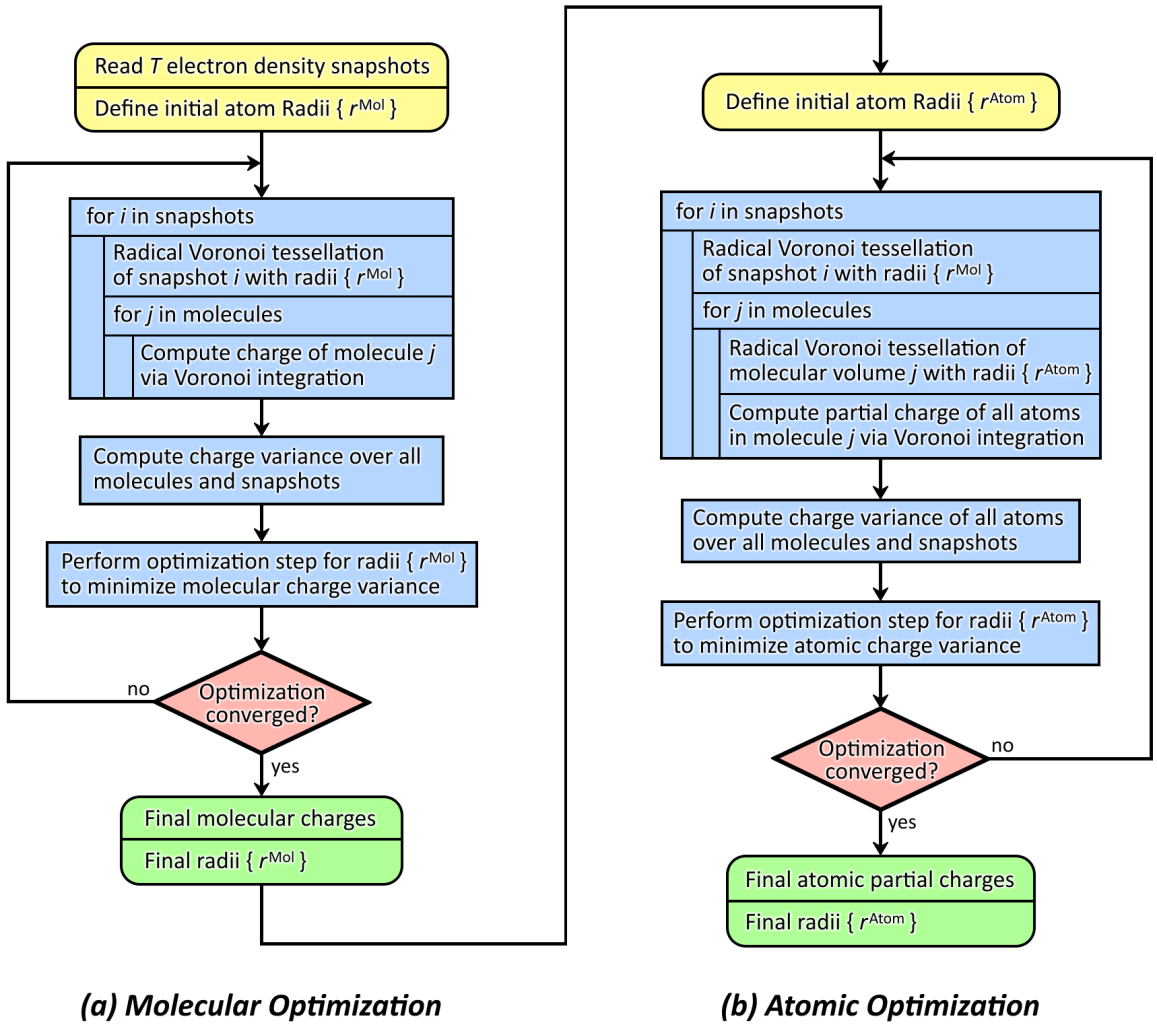
Flowchart of the proposed two-step optimization algorithm which yields optimized molecular and atomic partial charges as well as two sets of optimized atomic radii $\left\{r^{\mathrm{Mol}}\right\}$ and $\left\{r^{\mathrm{Atom}}\right\}$ via minimization of the charge variance.

Altogether, this leads to the schematic flowchart in Figure 1. We have implemented this algorithm in our open-source program package TRAVIS [70,71], thus it is freely available to the scientific community. The details of the implementation will be discussed in the following sections.

### 2.2. Voronoi Tessellation and Radical Voronoi Tessellation

The Voronoi tessellation [34] is a mathematical tool which partitions an Euclidean space containing some points (*Voronoi sites*) into non-overlapping subsets. Each Voronoi site corresponds to exactly one such subset (called *Voronoi cell*), which contains all points from space which are closer to this Voronoi site than to any other Voronoi site. In mathematical form, this is written as

(3) $$C_{i}:=\left\{x\in R^{n}|\;∥x-p_{i}∥\leq ∥x-p_{j}∥\;\forall j\in \left\{1\ldots k\right\},\;j\neq i\right\},\;i\in \left\{1\ldots k\right\},$$

where $R^{n}$ stands for any Euclidean space with the norm ∥·∥, in which *k* Voronoi sites, each with position $p_{i}\in R^{n}$, are given, and the $C_{i}\subseteq R^{n}$ are the resulting Voronoi cells.

By considering atoms in three-dimensional space as Voronoi sites, this concept has widely been applied in different fields of computational chemistry. To name a few advantages of the method, the Voronoi tessellation of a set of atoms is uniquely defined and can be calculated with moderate computational demands. The Voronoi tessellation can easily be adopted to systems with periodic boundary conditions, and is therefore well suited for bulk phase systems. Finally, the method does not possess any parameters to tune, and therefore gives an unbiased and uniquely defined picture.

However, certain limitations do arise from the properties of the standard Voronoi tessellation. As all atoms are treated in the same way, Voronoi polyhedra of light atoms like hydrogen will on average have the same size as those around heavier atoms like iodine. From a mathematical point of view, this is not a problem, but from a chemical perspective, this is completely unreasonable. If, e.g., the electron density within the Voronoi cell of a hydrogen atom is integrated, the hydrogen atom would always end up with a heavily negative partial charge, because way too much electron density would be considered as belonging to this hydrogen atom.

To overcome this problem, radii need to be introduced into the Voronoi tessellation, allowing to treat different atom types differently. Several ways to do so have been proposed (see the introduction). However, many of them suffer from problems such as the “vertex error” [42,44] (i.e., holes in the tessellation) or a lack of general applicability to arbitrary systems. Therefore, the **radical Voronoi tessellation** [44] (also termed “power diagram” in the two-dimensional case) will be employed here. It is an extension of the classical Voronoi tessellation, where a radius $r_{i}$ is assigned to each Voronoi site $p_{i}$. This results in the definition

(4) $$C_{i}^{r}:=\left\{x\in R^{n}|\;{∥x-p_{i}∥}^{2}-r_{i}^{2}\leq {∥x-p_{j}∥}^{2}-r_{j}^{2}\;\;\forall j\in \left\{1\ldots k\right\},\;j\neq i\right\},\;i\in \left\{1\ldots k\right\}.$$

**Figure 2:**
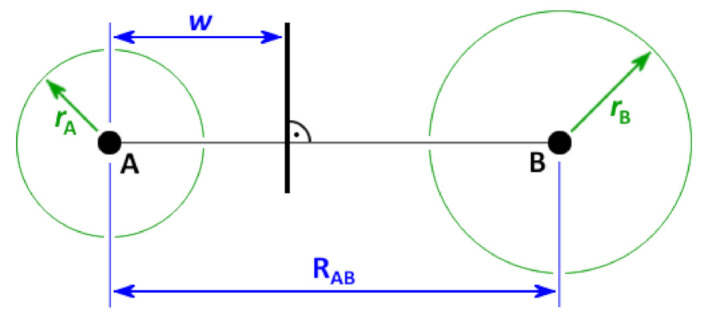
Separation plane between two Voronoi sites A and B with radii $r_{A}$ and $r_{B}$ in the radical Voronoi tessellation, see Equation (5).

**Figure 3:**
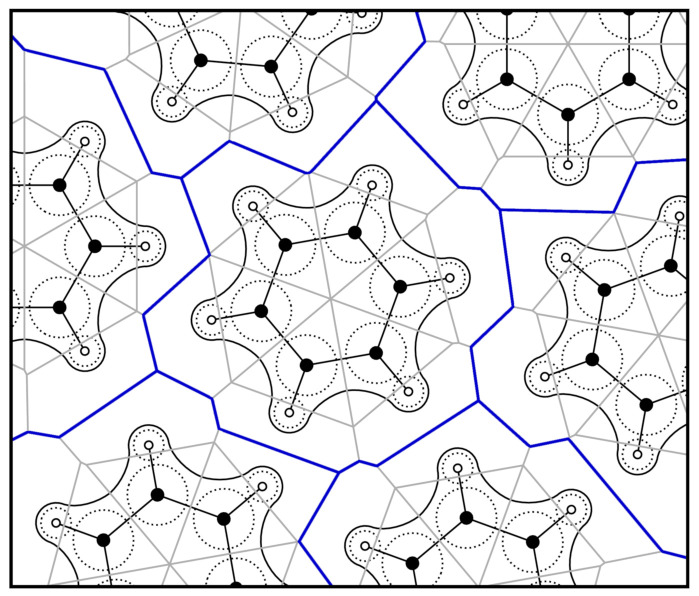
Schematic two-dimensional illustration of the radical Voronoi tessellation in the bulk phase of benzene. The solid black lines are iso-lines of the electron density, the dashed circles indicate the atomic radii, and the radical Voronoi cells are shown as gray solid lines with the resulting molecular boundaries drawn in blue. The position of the blue lines is exclusively determined by the first set of atomic radii $\left\{r^{\mathrm{Mol}}\right\}$, while the position of the gray lines is solely determined by the second set of radii $\left\{r^{\mathrm{Atom}}\right\}$.

While in the classical case the face between two adjacent Voronoi cells is always placed in the middle between the corresponding Voronoi sites, its position is now determined by the difference of the squared radii. From Equation (4), it can be derived that the separation plane between two sites A and B with radii $r_{A}$ and $r_{B}$ will be located at a position

(5) $$w:=\left(\frac{1}{2}+\frac{r_{A}^{2}-r_{B}^{2}}{2R_{\mathrm{AB}}^{2}}\right)R_{\mathrm{AB}},$$

where $R_{\mathrm{AB}}$ is the distance between both sites, and *w* describes the distance of the separation plane from A—see Figure 2. It can be seen that the relative position of the plane depends on the distance between the sites: if the distance becomes large with respect to the radii, the plane will be located in the middle, even if the radii differ. In the other extreme case of a small inter-site distance when compared to the radii, *w* can even be outside of the interval $\left[0,R_{\mathrm{AB}}\right]$, which either means that one of the sites is no longer located inside of its Voronoi cell, or the Voronoi cell of this site is degenerate (empty). However, both cases are not a problem if electron density shall be integrated within the cells. These effects are more pronounced if the differences between the radii become larger. If all radii are equal, the radical Voronoi tessellation becomes identical to the classical Voronoi tessellation, and those degeneracies cannot occur. A two-dimensional schematic illustration of the radical Voronoi tessellation in the case of benzene is shown in Figure 3. Please note that the term “radical” is not related to chemical radicals (which possess unpaired electrons).

The definition of the radical Voronoi tessellation in Equation (4) shows that the tessellation will not change if the set of radii $\left\{r_{i}\right\}$ is transformed to a new set $\left\{r_{i}^{\prime }\right\}$ by the map

(6) $$r_{i}^{\prime }:=\sqrt{r_{i}^{2}+C},\;i\in \left\{1\ldots k\right\}$$

with some constant C∈R. Due to this relation, the absolute value of the radii does not have a direct meaning, and one degree of freedom drops out of the optimization problem described below.

In the TRAVIS implementation of the method presented herein, the Voro++ library [72,73] from Chris Rycroft is used to perform the radical Voronoi tessellation of periodic simulation cells, which may have the shape of any parallelepiped (therefore not restricting our implementation to orthorhombic cells).

### 2.3. Integrating over Voronoi Cells

After the construction of the Voronoi cells, the electron density needs to be integrated within each Voronoi cell and the core charge has to be added to yield the partial charge of the corresponding atom. As the electron density in the simulation box is supplied on a grid, an efficient algorithm is required to traverse the grid points which are located inside a given Voronoi cell. A simplistic approach that checks for each grid point in which cell it is located would lead to very poor performance, as there are around 10 million grid points per snapshot (see Table 1). Instead, we have implemented another method: the three stride vectors of the grid are termed $v_{1},v_{2},v_{3}$ in the following. As non-orthorhombic simulation cells are permissible, these vectors do not need to be orthogonal to each other. At first, the maximum cross section of the Voronoi cell along the $v_{1}$ direction is computed in the $v_{2}$–$v_{3}$ plane. A (in the case of orthorhombic simulation cells) rectangular bounding box in that plane is constructed around this section. For each grid coordinate pair within this bounding box in the $v_{2}$–$v_{3}$ plane, a ray is cast into $v_{1}$ direction, and intersections between this ray and all Voronoi faces of the given Voronoi cell are probed. As Voronoi cells are always convex, there may be either zero or two such ray–face intersections, other combinations are not possible. With zero intersections, the ray misses the Voronoi cell, and no further action is taken. With two intersections, the entry and exit points of the ray through the Voronoi cell are known, and the grid points between the intersections can be summed up along the ray. This algorithm finally yields the sum over all grid points located within the given Voronoi cell. As each grid point is assigned to exactly one Voronoi cell by this algorithm, the total sum over all Voronoi cells is equal to the total sum over all grid points, which is important to keep the total charge of the system fixed. This implementation has already been applied several times to obtain the electric dipole moments of molecules in bulk phase simulations [45,52,53]. Our approach is rather efficient—a full Voronoi integration of a bulk phase snapshot with around 1000 atoms and 10 million grid points takes roughly 1 s on a single CPU core.

In real-world applications, the grid of the electron density is relatively coarse in order to reduce the required storage space for the volumetric data. Typical values are in the order of one grid point each 10…20 pm. As each grid point is completely assigned to exactly one Voronoi cell, infinitesimal changes in the radii may lead to grid points switching the cell they are assigned to. Therefore, the map from atomic radii to atomic charges is no longer continuous, or in other words, some amount of numerical discretization noise is introduced, which hampers the optimization algorithm for the radii. To reduce the impact of this effect, we have developed and implemented an on-the-fly interpolation scheme for the electron density grid. During the integration pass, the grid can be refined via tri-linear interpolation. The smaller grid spacing which results from this procedure leads to a reduced amount of numerical noise. On the other hand, demands on storage system and core memory are not increased, as the interpolation is just performed on-the-fly while integrating. We call this approach *refinement*; it has been utilized in all applications of our method presented herein with a refinement factor of 2 (i.e., one grid point was interpolated to two grid points along each axis of the grid, yielding 8 grid points in total from each original grid point). Our implementation is not limited to a refinement factor of 2; higher values can be chosen on demand.

### 2.4. Charge Variance Minimization Algorithm

As the objective function of the optimization problem to be solved is the charge variance in dependence of the atomic radii for the radical Voronoi tessellation of an arbitrarily complex chemical system, it is easy to see that analytic expressions for the first derivatives or for the Hessian of the objective with respect to the radii are not available. Therefore, the gradient needs to be determined by numerical differentiation with respect to all radii by following a finite-difference scheme. Unfortunately, the objective function is not strictly continuous due to numerical noise introduced by the Voronoi tessellation and the discrete grid of the electron density (see Section 2.3). A simple one- or two-sided finite difference approach therefore did not yield reliable gradients. Thus, our current implementation takes three equidistant samples of the objective function on each side, giving a total amount of seven samples (including the central position). A linear regression is performed on these points, and the point which deviates most from the regression equation is removed. On the remaining six points, another linear regression is performed, and the slope of the resulting function is used as the gradient along this direction. This procedure is repeated for each parameter of the optimization problem, i.e., each type of non-equivalent atoms. The maximum displacement of each parameter was chosen to be 0.15 pm in each direction.

Based on this algorithm for the gradient calculation, a numerical optimization scheme can be set up. We have decided to utilize the non-linear conjugate gradient (CG) method [74] in combination with golden-section bracketing line search [75,76] due to the robustness and resilience of this approach. There exist several flavors of the nonlinear conjugate gradient method. Our implementation utilizes the Polak–Ribiére formula [77,78] to obtain the CG mixing parameter β according to

(7) $$\beta_{k}:=\frac{r_{k+1}^{T}\left(r_{k+1}-r_{k}\right)}{r_{k}^{T}r_{k}},$$

which yields better convergence rates than the original Fletcher–Reeves formula [74] in many cases. If the value of β becomes negative, the usual approach to perform a downhill step instead in order to reset the conjugate-gradient scheme has been implemented. Due to the strong numerical noise, another measure is necessary: If the line search returns a step which would increase the objective, this step is rejected, and the line search bracketing is repeated with a reduced initial search interval. If the search interval falls below some small threshold, a downhill step with line search is attempted. If this still does not reduce the objective for more than a given threshold, the optimization is considered to be converged. As initial radii for the optimization, we used van der Waals element radii [46,47,48] for $\left\{r^{\mathrm{Mol}}\right\}$ and covalent element radii [79] for $\left\{r^{\mathrm{Atom}}\right\}$. Since these radii are expected to yield already a reasonable partition of the electron density between molecules and atoms, respectively, the optimal radii should not be vastly different. This choice therefore reduces the number of optimization steps and thus the required computer time. For an optimization problem which possesses only one minimum, the choice of initial values does not influence the results at all. For the simple example of methanol, it is discussed in Section 3.1 and visualized in Figure 5 that there is only one charge variance minimum for the separation of the molecular electron density, which is not far from the van der Waals radii.

## 3. Results and Discussion

**Figure 4:**
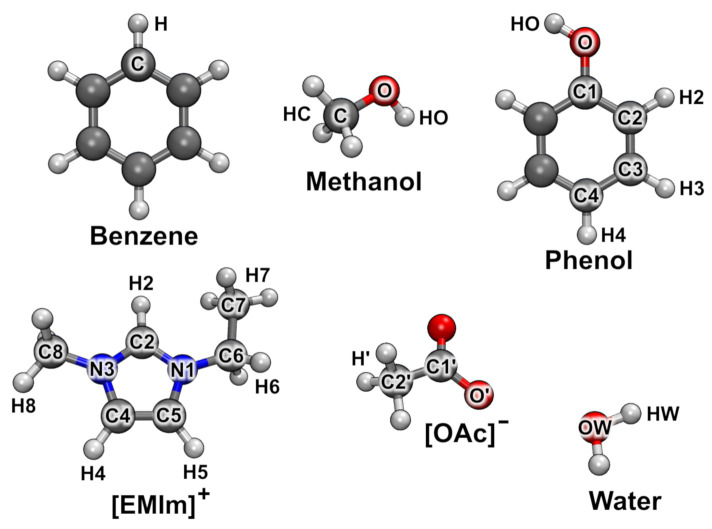
Atom labeling of the molecules studied. Element colors: gray—carbon, red—oxygen, blue—nitrogen, white—hydrogen.

In order to discuss some results of our proposed method, it has been applied to several ab initio molecular dynamics simulations. Five trajectories of different liquid phase simulations are evaluated within this work, see Table 1. The last two systems contain ion pairs of the ionic liquid 1-ethyl-3-methylimidazolium acetate, abbreviated as [EMIm][OAc]. These two trajectories have been published and discussed in the literature before [50,80,81,82]. The atom nomenclature within the studied molecules is described in Figure 4. In the following, the results from the analysis of the five example systems are presented.

### 3.1. Molecular Charges

**Figure 5:**
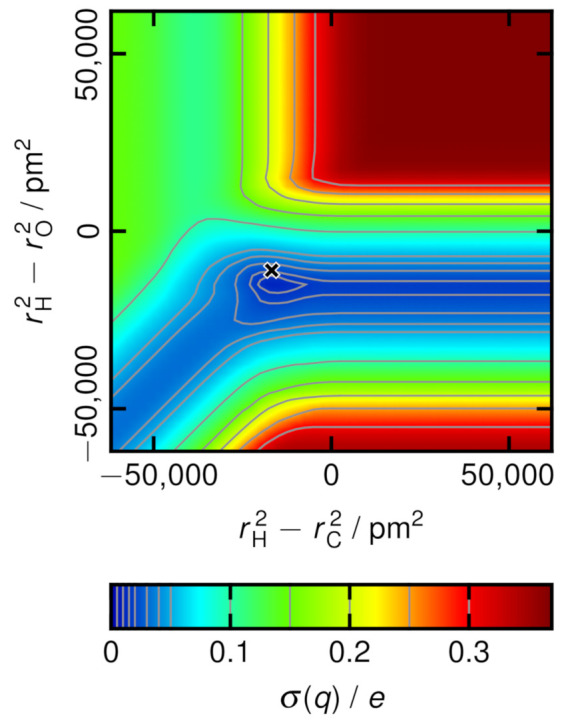
Standard deviation of the charge distribution in the methanol system as a function of the Voronoi radii (assuming $r_{\mathrm{HC}}=r_{\mathrm{HO}}=r_{H}$ here). The σ values are given in units of *e*. The black cross marks the van der Waals radii [46,47,48].

To visualize the optimization problem in the case of molecular charge fluctuations, a contour plot of the standard deviation of the molecular charge in dependence of the Voronoi radii is given in Figure 5 for the methanol system. In methanol, there are four different types of atoms, and therefore, three degrees of freedom remain after factoring out the invariance from Equation (6), namely the three differences $r_{\mathrm{HC}}^{2}-r_{\mathrm{HO}}^{2}$, $r_{\mathrm{HC}}^{2}-r_{C}^{2}$, and $r_{\mathrm{HC}}^{2}-r_{O}^{2}$. In order to arrive at a two-dimensional function for the sake of illustration, the additional constraint $r_{\mathrm{HC}}=r_{\mathrm{HO}}=r_{H}$ has been applied here, which only concerns the contour plot, and is in contrast to the computations performed in the remainder of this section, were $r_{\mathrm{HC}}$ and $r_{\mathrm{HO}}$ are independent parameters. This leaves only two differences of squared radii as degrees of freedom. The plot in Figure 5 shows that there exists a well-defined minimum of the standard deviation of the molecular charge, which is close to the van der Waals radii of the atoms, as it is already expected from the discussion in the introduction. The point in the origin of the plot—which corresponds to the classical non-radical Voronoi tessellation—possesses a significantly larger molecular charge fluctuation. For large absolute values of the differences, stationary states are reached where no further changes appear. In these situations, the Voronoi cells of the atoms with the smaller radius completely vanish, so these atoms do no longer appear in the tessellation at all. In the upper right corner of the plot, only the hydrogen atoms possess a Voronoi cell, while only the carbon atoms remain in the upper left corner, and the tessellation just contains the oxygen atoms in the lower right corner. Consequently, the carbon atoms disappear in the valley to the right side, the oxygen atoms vanish in the valley to the top, and the hydrogen atoms do not have a cell in the valley to the lower left corner. The deepest valley is the one leading to the right side, indicating that it is most important to correctly place the cell faces between hydrogen and oxygen atoms for a reasonable distribution of the electron density to the methanol molecules.

The molecular charges that were obtained by applying the proposed optimization algorithm are given in Table 2. For the non-ionic systems which contain only one molecule type (benzene, methanol, phenol), it is clear by definition that the average molecular charge always has to be zero. For these systems, only the standard deviation of the charge is of interest. We find that the standard deviation of the molecular charge is quite small in all cases, in the range of 0.01 *e*. This indicates that the electron density is well partitioned to the individual molecules, so that each molecule is close to its expected average charge of zero in every configuration.

When considering the pure ionic liquid system (IL), all observations concerning the standard deviation of molecular charges from above still hold. More interesting, the ionic charges are found to be within a range of 0.8e…0.85e. It has been shown before that ion charges of approximately 0.8e lead to a good reproduction of experimental quantities in classical molecular dynamics simulations [1,2,56,57]. In many force field molecular dynamics studies of ionic liquids up to now, the ion charges have been empirically scaled down to achieve this effect [1,2,3,57,60,61,62], which is no longer required with our approach. The finding that average charges of anion and cation equal each other in absolute value follows by definition from the charge neutrality of the simulation box.

Finally, there are some interesting points to note for the ionic liquid/water mixture (ILW). The charge neutrality of the system is also fulfilled in this case—please note that the average ionic/molecular charges need to be multiplied with the number of ions/molecules in order to sum up to zero (see Table 1). The cation charge becomes larger by the addition of water, whereas the anion charge becomes smaller in absolute value. Therefore, the absolute value of the cation charge is on average larger than that of the anion charge, and the water molecules possess a small negative charge on average (however, smaller than the standard deviation in absolute value). These findings indicate that the water molecules interact more strongly with the anions than with the cations, as this explains both observations: Cations become even more positive due to missing hydrogen bonds to the acetate, as most acetate hydrogen bond acceptors are occupied by water molecules now. Acetate anions become less negative on average, as they can form much more and stronger hydrogen bonds to water molecules *(imidazolium cations are fairly weak hydrogen bond donors in contrast to water)*. These relations have already been observed in earlier articles about this ionic liquid by using a different methodology [62,80,81]. Thus, the molecular charges obtained by our method even provide some insights into the chemistry and binding affinity of the compounds of a mixture.

### 3.2. Atomic Partial Charges and Radii

In this section, the atomic partial charges as well as the two sets of optimized radii $r^{\mathrm{Mol}}$ and $r^{\mathrm{Atom}}$ which have been obtained from the analysis of the example systems will be discussed, see Table 3 and Table 4 *(the results are split over two tables for better readability)*.

In case of the benzene system, there are only two non-equivalent atom types. Much electron density of the benzene molecules is assigned to the carbon atoms, so the hydrogen atoms bear a positive charge of around +0.14e. The standard deviation of the atomic charges is around 0.01 *e* and therefore quite small, indicating a reasonable partitioning of the electron density to the individual atoms. Considering the optimized radii, we find that hydrogen possesses a significantly smaller radius than carbon in both sets, as it would be expected from chemical intuition. The radii from molecular charge optimization are larger than those from atomic charge optimization, similarly to van der Waals radii [46,47,48] being larger than covalent radii [79].

Concerning the methanol simulation, it can be seen that the oxygen atom bears a significantly negative charge of −0.61e, while the hydroxyl proton is positively charged with +0.35e, leading to a strong dipole in the hydroxyl group as expected. The central carbon atom is very positive with a charge of +0.76e, while the aliphatic protons are negative at around −0.17e. The standard deviation in the charges of the carbon and aliphatic hydrogen atoms is small, while that of the oxygen and hydrogen atoms from the hydroxyl group is larger by a factor of 2…3. This is due to the strong hydrogen bond which can be formed between hydroxyl groups: if a hydrogen bond is present, the atomic charges will be different due to a certain amount of charge transfer, so that the standard deviation is larger. An increased standard deviation of an atomic charges is therefore an indication for a strong directed interaction involving this atom. For the atom radii, we find that the hydroxyl proton possesses by far the smallest value, significantly smaller than that for the aliphatic hydrogen atoms. This nicely shows how our approach assigns different radii to atoms of the same element, depending on their chemical environment and bonding situation. It also confirms our decision to vary the radii of non-equivalent atoms individually in the optimization procedure *(rather than treating all atoms of one element with the same radius)*.

For the phenol simulation, the picture becomes slightly more complex, as we have nine non-equivalent types of atoms now. As expected, the hydroxyl oxygen atom bears a significantly negative charge of −0.45e, while the hydroxyl proton is positive with +0.23e. The aromatic C1 carbon atom is significantly positive due to the electron-withdrawing effect of the hydroxyl group. It is very interesting to note that we find an alternating “positive–negative–positive–negative” charge pattern *(relatively to the average carbon charge of +0.14e)* when going from C1 to C4 which attenuates with rising distance from the hydroxyl group. This alternating pattern is well known in aromatic chains and rings, and is an important cause of the regioselectivity in aromatic reactions. Also here, the standard deviation of the oxygen and hydrogen atom of the hydroxyl group is significantly larger than the value of all other atoms, indicating a strong directed interaction, which is again a hydrogen bond.

In the pure ionic liquid system (IL), some interesting observations can be made. The most positive hydrogen atom is the central ring proton H2, which is known to be a hydrogen bond donor [62,80,81,82,83,84]. The other two ring protons H4 and H5 are significantly less positive. H5 even is assigned a slight negative charge of −0.09e, which certainly is an unexpected result. A significant amount of the positive charge is concentrated in the two side chains of the ion, while the ring only possesses a slight positive charge of around +0.1e in total. In acetate, the oxygen atoms bear a strong negative charge as expected, while the carboxyl carbon atom is significantly positive. Concerning the standard deviation of the atomic charges, the observation from above holds: strong hydrogen bonds exist between the ring protons H2, H4, and H5 and the acetate oxygen atoms O’, and these four atoms also possess the largest standard deviation in their charge due to the charge transfer effect of the hydrogen bond.

When going to the mixture of the ionic liquid with water (ILW), most observations from the last paragraph still hold. The central ring proton H2 becomes slightly less positive when water is added (i.e., *water depolarizes the [EMIm]${}^{+}$ cation*), while the acetate oxygen atoms become even more negative due to the water addition (i.e., *water polarizes the [OAc]${}^{-}$ anion*). This is exactly what was observed in literature before from AIMD simulations of this mixture [80]. It is interesting to note that the charge distribution within the water molecules yields $q_{\mathrm{OW}}=-0.55\;e$, which indicates a slightly weaker charge separation than in popular three-site water force fields, like, e.g., $q_{\mathrm{OW}}=-0.85\;e$ in SPC/E [85]. This might be due to the fact that the hydrogen bond network of water is interrupted by the ionic liquid—in particular by the almost non-polar cation—so that water molecules become depolarized in the vicinity of [EMIm]${}^{+}$ as observed before [80].

Based on these considerations, it can be stated that the atomic charges and radii obtained by our two-step approach are helpful for understanding the relevant interactions in liquid-phase systems. Strong directed interactions can be identified by an increased standard deviation of the atomic charge due to the charge transfer effect. In phenol, the alternating charge pattern induced by the hydroxyl substituent could be well observed, so that our charges are able to predict reactivity and regioselectivity in aromatic systems. Finally, the optimized atomic radii show a significant distinction between atoms of the same element—for example, protic hydrogen atoms such as the [EMIm]${}^{+}$ ring protons and the hydroxyl protons are assigned a smaller radius than aliphatic hydrogen atoms, and can thus be identified in a bulk phase simulation. Therefore, our optimized radii are more flexible than van der Waals [46,47,48] or covalent [79] atom radii from literature, which possess fixed values per element.

In order to show that our two-step Voronoi integration scheme with optimized radii is indeed worth the effort, we have computed the Voronoi charges for the five systems from Table 1 of the manuscript via a one-step Voronoi integration with three different sets of non-optimized standard radii. The results are presented in Tables S1–S4 in the Supplementary Materials, where we also discuss the values. In summary, we demonstrate there that a single set of radii in the radical Voronoi tessellation is not able to describe both the separation between individual molecules and the partitioning of the molecular electron density to the atoms well at the same time. If both shall be described in a reasonable way, two different sets of radii need to be employed simultaneously, as we do in our two-step approach which is presented here.

### 3.3. Basis Set Dependence

When considering methods for computing atomic partial charges via electron structure theory, a very important quantity is the basis set dependence of the method. While methods which directly evaluate the molecular orbitals *(such as Mulliken and Löwdin charges)* are typically very sensitive with respect to the choice of the basis set, methods which only rely on the total electron density *(such as RESP and Hirshfeld charges)* are often more robust and show less dependence.

To investigate the basis set dependence of our approach, we have re-calculated the snapshots for the ILW system with three different basis set sizes (MOLOPT-SZV-SR, MOLOPT-DZVP-SR, MOLOPT-TZVPP-SR) [86] and used the electron densities resulting from these calculations to compute our two-step Voronoi charges. The size of the three basis sets differs considerably. While the SZV basis possesses only 0.87 basis functions per electron in the case of the ILW system, the DZVP basis set possesses 3.21 functions per electron, and the TZVPP basis set even 5.54 functions per electron for this system (13,770 functions in total). As all our calculations are restricted Kohn–Sham calculations, electrons are considered as pairs, so that values of less than one basis function per electron are possible *(a minimal basis set would have 0.5 functions per electron in an RKS calculation)*.

**Figure 6:**
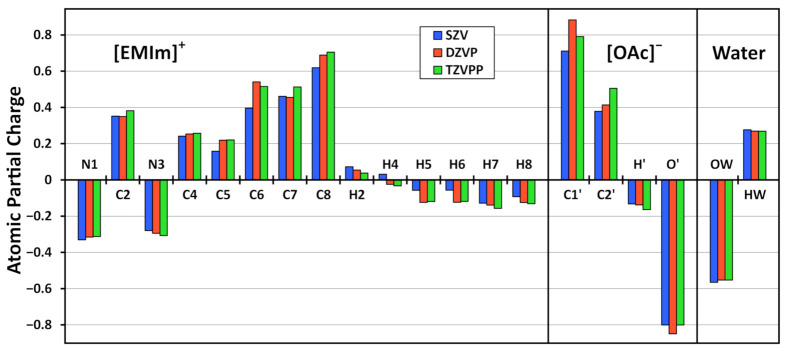
Influence of the basis set size on the optimized atomic partial charges in the ILW simulation. Vertical axis in units of *e*. For atom labels, see Figure 4.

The results from our calculations are presented in Table 5 and as a diagram in Figure 6. At first sight, it can be seen that the basis set dependence is relatively small, and the optimized atomic partial charges qualitatively agree with all three basis sets. When going from SZV to DZVP, there are slightly larger changes for some atom types. This can be easily understood from the fact that the SZV basis set is tiny (with only 0.87 basis functions per electron), and is just not able to describe the electron structure of the system in a reasonable way. Between DZVP and TZVPP, the agreement is better, except for the two carbon atoms of acetate C1’ and C2’. When comparing SZV to TZVPP, the average deviation between atomic charges is 0.04e, while for DZVP to TZVPP it is only 0.02e. This is in the range of the standard deviation of the atomic charges obtained by our approach (see Table 3 and Table 4), and therefore completely satisfactory. We can therefore conclude that the basis set dependence of our two-step Voronoi charge approach is small, similar to other methods for computing charges which only rely on the total electron density.

## 4. Computational Details

All simulations have been carried out with the program package CP2k [87,88,89], using the Quickstep module [90] in conjunction with the orbital transformation (OT) algorithm [91]. The electron structure was calculated by density functional theory [92,93], using the BLYP functional [94,95] together with the recent re-parametrization [96] of Grimme’s D3 dispersion correction [97,98] with Becke–Johnson damping. Basis sets of the kind DZVP-MOLOPT-SR [86] together with Goedecker–Teter–Hutter (GTH) pseudopotentials [99,100,101] have been applied. The plane wave cutoff was set to 350 Ry with a REL_CUTOFF of 40. The temperature during the simulations was kept constant using a Nosé–Hoover chain thermostat [102,103,104,105] with a time constant of 100 fs. The integration time step was set to 0.5 fs in all cases.

For the benzene, methanol, and phenol systems, the initial configurations were created with the Packmol software [106]. A force field pre-equilibration with OPLS–AA force field parameters [107] was performed using LAMMPS [108]. These three simulations have been equilibrated for some picoseconds with massive thermostating before the start of the production run. The IL and ILW systems were restarted from the last snapshot of the AIMD trajectories which were already published in literature [80,81,82]. Please note that [EMIm][OAc] refers to the ionic liquid 1-ethyl-3-methylimidazolium acetate [80,81,82].

The electron densities of the simulation cells have been written to disk as volumetric data with a grid spacing of approximately 10 pm, yielding typical grid resolutions of around 240×240×240 for the large systems. A snapshot of the electron density was written every 1000 time steps (i.e., every 0.5 ps) and compressed in our recently published bqb format [109], which is now directly available in CP2k and yields a lossless compression ratio of around 40:1, so that the storage requirements became tractable. 32 such snapshots have been evaluated for each system, sampling 16 ps of physical time. An on-the-fly refinement factor of 2 (see Section 2.3 above) has been used for integrating the Voronoi cells for all five systems. The determination of the factor value was based on the observation that the charges seem to be well converged with a factor 2 in our applications, and larger values no longer lead to significant changes in the results.

Figure 4 was created with VMD [110] and Tachyon [111], while Figure 5 has been prepared with Gnuplot [112].

## 5. Conclusions

In this article, we presented a novel method for the calculation of optimized molecular charges and atomic partial charges from bulk phase simulations, which is based on a two-step radical Voronoi tessellation of the system and subsequent integration of the electron density within each Voronoi cell. First, the total electron density is partitioned into the contributions of each molecule by using a set of atomic radii $\left\{r^{\mathrm{Mol}}\right\}$, while in the second step, the electron density within each molecule is assigned to the individual atoms using a second set of atomic radii $\left\{r^{\mathrm{Atom}}\right\}$. Both sets of radii are optimized on-the-fly to minimize charge fluctuations of atoms and molecules. Therefore, our approach yields atomic partial charges without any empirical parameters. Two sets of optimized atomic radii are obtained as a by-product from each run. It should be noted that the absolute values of these radii do not have a direct meaning, since only the differences of squared radii determine the partitioning of the total space into molecular volumes and the partitioning of each molecular volume into atomic cells. Nevertheless, these two sets of radii can be considered specialized van der Waals and covalent radii, which resemble many properties and interactions in the specific system investigated, and can be applied for further analysis. The ability to handle periodic systems—including non-orthorhombic cells—makes the method particularly well suited for the application to bulk phase systems such as simulations of liquids. Only the total electron density distribution on a grid is required as input data. Therefore, our method is not limited to Hartree–Fock or DFT, and can be easily combined with all electronic structure methods that are able to provide the total electron density on a real-space grid, such as orbital-free approaches [113], DFTB [114,115,116,117,118], multi-reference calculations, or even MP2 [119,120] and coupled-cluster methods [121,122,123]. With our recently published lossless compression algorithm for electron density trajectories (“bqb format”, [109]) which yields a lossless compression ratio of around 40:1, the storage requirements of this data along a simulation trajectory become tractable.

In the second part of the article, we applied the newly developed method to five bulk phase simulations of organic molecules and ions and discussed the results. The standard deviations of the molecular and atomic charges are around 0.01e and therefore very small, which indicates a reasonable partitioning of the total electron density to the molecules and atoms. We show that the optimized charges and radii obtained by our approach are useful to understand the interactions in the system. For example, strong directed interactions such as hydrogen bonds can be detected by an increased charge standard deviation of the involved atoms. In the case of phenol, we find the typical alternating charge pattern in the aromatic system that is induced by the hydroxyl substituent, so that our charges can even be used to predict reactivity and regioselectivity in aromatics. From the application to bulk phase ionic liquid systems, it was shown that the well-known reduction of ionic charge below unity [1,2,3,56,57,58,59] is reproduced, predicting ion charges in the range of 0.8…0.85e without any empiricism, tuning, or constraints. In many force field molecular dynamics studies of ionic liquids up to now, the ion charges have been empirically scaled down to achieve this effect [1,2,60,61,62], which is required to obtain the right time scale for the dynamics. However, it has been recently shown that a simple scaling-down procedure of atomic charges might break the subtle balance of interactions within such complex systems [63]. With our approach, such a down-scaling is unnecessary, as the resulting ion charges are already within the range of values that have been shown do yield realistic dynamics [1,2,3,56,57,58,59]. Apart from that, we have demonstrated that the use of either van der Waals or covalent radii in a one-step Voronoi integration (using only one set of radii) does not yield reasonable results, and our two-step approach is indeed necessary (see Supporting Information).

In the last part of the study, we investigated the basis set dependence of our method, and find it to be very small, as it is expected from a method that only utilized the total electron density. When going from a DZVP to a TZVPP basis set, the resulting atomic partial charges show a deviation of only 0.02e on average.

## Figures and Tables

**Table 1:** Exemplary liquid phase simulations to which the method is applied, including composition, orthorhombic cell vector, density, simulation temperature, and total electron density volumetric grid resolution.

System	Composition	Cell/pm	Density/g cm${}^{-3}$	Temp./K	Grid Resolution
Benzene	32 benzene	1690	0.860	350	192×192×192
Methanol	48 methanol	1515	0.735	350	180×180×180
Phenol	32 phenol	1688	1.040	400	192×192×192
IL	36 [EMIm]^+^ 36 [OAc]^−^	2121	1.066	350	240×240×240
ILW	27 [EMIm]^+^ 27 [OAc]^−^ 81 H_2_O	2158	1.000	350	243×243×243

**Table 2:** Resulting average molecular charges and corresponding standard deviation for the five simulations from Table 1. All numbers in units of *e*.

Molecule	Charge	Std. Dev.
Benzene	0	0.010
Methanol	0	0.013
Phenol	0	0.012
**IL**		
[EMIm]${}^{+}$	0.846	0.016
[OAc]${}^{-}$	−0.846	0.011
**ILW**		
[EMIm]${}^{+}$	0.859	0.017
[OAc]${}^{-}$	−0.817	0.015
Water	−0.014	0.015

**Table 3:** Resulting atomic partial charges, standard deviations as well as molecular and atomic Voronoi radii for the first three systems from Table 1. Charges and standard deviations in units of *e*, radii in pm. For atom labels, see Figure 4.

Atom	Charge	Std. Dev.	$r^{\mathrm{Mol}}$	$r^{\mathrm{Atom}}$
**Benzene**				
C	0.141	0.007	173.7	72.9
H	−0.141	0.010	107.6	41.8
**Methanol**				
C	0.764	0.008	172.5	76.4
HC	−0.169	0.010	112.9	51.7
O	−0.609	0.023	152.9	81.1
HO	0.350	0.026	104.0	28.8
**Phenol**				
C1	0.248	0.014	170.1	73.8
C2	0.084	0.013	172.8	73.9
C3	0.154	0.010	171.6	72.9
C4	0.122	0.012	173.2	72.8
H2	−0.115	0.011	107.9	41.3
H3	−0.134	0.011	111.5	41.5
H4	−0.130	0.011	111.4	40.4
O	−0.447	0.025	152.9	78.0
HO	0.229	0.024	104.3	39.2

**Table 4:** Resulting atomic partial charges, standard deviations as well as molecular and atomic Voronoi radii for the final two systems from Table 1. Charges and standard deviations in units of *e*, radii in pm. For atom labels, see Figure 4.

Atom	IL				ILW			
	Charge	Std. Dev.	$r^{\mathrm{Mol}}$	$r^{\mathrm{Atom}}$	Charge	Std. Dev.	$r^{\mathrm{Mol}}$	$r^{\mathrm{Atom}}$
**[EMIm]${}^{+}$**								
N1	−0.306	0.011	167.3	82.7	−0.316	0.011	162.2	80.8
C2	0.270	0.015	165.2	79.5	0.350	0.015	160.1	76.9
N3	−0.223	0.013	163.7	80.3	−0.294	0.011	168.3	80.1
C4	0.149	0.014	169.1	78.0	0.254	0.014	168.1	74.8
C5	0.148	0.013	169.1	78.7	0.219	0.013	171.9	75.7
C6	0.468	0.009	172.3	73.5	0.541	0.008	170.3	71.7
C7	0.518	0.007	171.4	71.4	0.455	0.007	178.0	71.9
C8	0.655	0.010	165.2	72.0	0.688	0.008	163.5	71.4
H2	0.092	0.025	107.3	37.3	0.055	0.022	108.0	36.3
H4	0.041	0.022	106.9	38.2	−0.025	0.018	105.1	40.8
H5	−0.093	0.019	107.3	39.8	−0.124	0.015	109.8	40.8
H6	−0.092	0.020	107.3	39.8	−0.123	0.015	109.8	40.8
H7	−0.156	0.015	109.6	41.6	−0.139	0.014	105.9	40.2
H8	−0.115	0.019	108.9	41.7	−0.125	0.014	110.1	42.1
**[OAc]${}^{-}$**									
C1’	0.761	0.015	162.4	73.4	0.882	0.018	165.2	72.7
C2’	0.482	0.008	175.2	71.7	0.414	0.008	175.2	73.5
H’	−0.162	0.011	109.8	41.1	−0.138	0.011	109.1	41.8
O’	−0.801	0.020	155.5	83.1	−0.849	0.024	155.6	85.0
**Water**									
OW					−0.553	0.024	155.8	79.2
HW					0.269	0.025	108.1	36.0

**Table 5:** Optimized molecular and atomic partial charges as well as corresponding standard deviations for the ILW simulation with three different basis set sizes. All numbers in units of *e*. For atom labels, see Figure 4.

Atom	SZV		DZVP		TZVPP	
	Charge	Std. Dev.	Charge	Std. Dev.	Charge	Std. Dev.
**[EMIm]${}^{+}$**	0.948	0.015	0.859	0.017	0.841	0.018
N1	−0.331	0.010	−0.316	0.011	−0.313	0.011
C2	0.352	0.015	0.350	0.015	0.382	0.015
N3	−0.280	0.011	−0.294	0.011	−0.307	0.011
C4	0.241	0.014	0.254	0.014	0.257	0.014
C5	0.158	0.014	0.219	0.013	0.220	0.013
C6	0.395	0.009	0.541	0.008	0.515	0.008
C7	0.461	0.008	0.455	0.007	0.513	0.007
C8	0.619	0.009	0.688	0.008	0.705	0.008
H2	0.072	0.022	0.055	0.022	0.038	0.021
H4	0.031	0.019	−0.025	0.018	−0.033	0.018
H5	−0.057	0.014	−0.124	0.015	−0.119	0.015
H6	−0.057	0.014	−0.123	0.015	−0.118	0.015
H7	−0.128	0.013	−0.139	0.014	−0.157	0.014
H8	−0.093	0.013	−0.125	0.014	−0.131	0.014
**[OAc]${}^{-}$**	−0.909	0.016	−0.817	0.015	−0.797	0.015
C1’	0.711	0.018	0.882	0.018	0.791	0.015
C2’	0.378	0.009	0.414	0.008	0.506	0.008
H’	−0.132	0.010	−0.138	0.011	−0.164	0.011
O’	−0.800	0.028	−0.849	0.024	−0.801	0.024
**Water**	−0.013	0.014	−0.014	0.015	−0.015	0.015
OW	−0.565	0.027	−0.553	0.024	−0.553	0.023
HW	0.276	0.029	0.269	0.025	0.269	0.025

## Data Availability

The data presented in this study are available on request from the corresponding author.

## Supplementary Materials

The following are available online, Tables S1–S4: Molecular and atomic charges from non-optimized radii and a one-step Voronoi integration.

## Abbreviations

The following abbreviations are used in this manuscript:

[EMIm]${}^{+}$	1-Ethyl-3-methylimidazolium cation
[OAc]${}^{-}$	Acetate anion
BLYP	Becke–Lee–Yang–Parr density functional
bqb	The bqb file format for lossless compression
CG	Conjugate gradient optimization method
DDAPC	Density derived atomic point charges (Blöchl charges)
DFT	Density functional theory
DFTB	Density functional based tight binding
GTH	Goedecker–Teter–Hutter pseudopotentials
IL	Ionic liquid
MP2	Møller–Plesset perturbation theory
RESP	Restrained electrostatic potential
ROA	Raman optical activity
VCD	Vibrational circular dichroism
