# Supplementary material for: Optimized Atomic Partial Charges and Radii Defined by Radical Voronoi Tessellation of Bulk Phase Simulations
Source: Molecules. 2021 Mar 26;26(7):1875. doi: 10.3390/molecules26071875 (PMC8036805; doi:10.3390/molecules26071875)
Supplement: Supplementary file 1 [file molecules-26-01875-s001.pdf]

Optimized Atomic Partial Charges and Radii Defined by Radical Voronoi Tessellation of Bulk Phase Simulations

Supporting Information

Martin Brehm* and Martin Thomas

Institut für Chemie - Theoretische Chemie, Martin-Luther-Universität Halle-Wittenberg, Von-Danckelmann-Platz 4, 06120 Halle (Saale), Germany.

*E-mail: Martin_Brehm@gmx.de

*Website: https://brehm-research.de/

Comparison to One-Step Voronoi Integration

In order to show that our two-step Voronoi integration scheme with optimized radii is indeed worth the effort, we have computed the Voronoi charges for the five systems from Table 1 of the manuscript via a one-step Voronoi integration with three different sets of non-optimized standard radii. The results can be seen in Tables S1 to S4 below. The first set of radii which we considered are unity radii, *i. e.*, all atoms are assigned identical radii. In this case, the radical Voronoi tessellation becomes identical to the classical non-radical Voronoi tessellation (see Equations 3 and 4 in the manuscript). It is well known that the classical Voronoi tessellation does not lead to a reasonable partitioning of the electron density, because the size of hydrogen atoms is significantly over-estimated, so that they are assigned too much electron density and become heavily negatively charged. This is also observed here. The charge of hydrogen atoms in our simulations is found to be between $-0.4\,e$ and $-0.5\,e$ with unity radii. On the other hand, the oxygen atoms in methanol and phenol carry a positive charge of around $+0.25\,e$, which clearly contradicts chemical intuition. The ion charges in the IL system are predicted to be only $\pm 0.4\,e$. Furthermore, the standard deviation of the molecular charges is quite large (around $0.1\,e$). It can be concluded that the classical Voronoi tessellation without radii expectedly fails to yield reasonable molecular and atomic charges.

In the next step, we have performed a one-step Voronoi integration with van der Waals element radii from the literature.[1–3] As discussed above, these have already proven to lead to a reasonable partitioning of the space into molecular volumes. This becomes visible when considering the standard deviation of the molecular charges, which is within $0.01\,e \ldots 0.02\,e$, and therefore almost as low as with the optimized radii above. The ionic charges in the IL system are predicted to be $\pm 0.8\,e$, which seems reasonable. However, the atomic charges are all very large in absolute value—for example, the carbon atom in methanol bears a charge of $-2.5\,e$ with these radii, which is beyond any reasoning. Apart from that, the standard deviation of the atomic charges is quite large, which is another indication of a bad partitioning within the molecules. We conclude that van der Waals radii perform relatively well in distributing the electron density to the individual molecules, but completely fail to assign the electron density to the atoms within each molecule.

Finally, we used covalent element radii from the literature[4] to obtain charges from a one-step Voronoi integration. As these charges are based on covalent bond lengths, it can be expected that they are well suitable to describe the partitioning of electron density within each molecule to the individual atoms. The standard deviation of the atomic charges is relatively low with these radii, and also the values of the charges are in a reasonable range *(oxygen atoms with a negative charge, carbon atoms and hydroxyl protons with a positive charge, etc.)*. However, the molecular charges are of poor quality, as indicated by a large standard deviation. The ionic charge in the IL system is predicted to be $\pm 0.58\,e$, which is too small in absolute value. It turns out that the covalent radii are not well suited to describe the boundaries between individual molecules, where no covalent bonds are present.

In summary, we have shown that a single set of radii in the radical Voronoi tessellation is not able to describe both the separation between individual molecules and the partitioning of the molecular electron density to the atoms well at the same time. If both shall be described in a reasonable way, two different sets of radii need to be employed simultaneously, as we do in our two-step approach which is presented here.

Table S1: Average molecular charges and corresponding standard deviation for the five simulations from Table 1 in the manuscript, using three different sets of non-optimized standard radii and a one-step Voronoi integration. All numbers in units of e.

	**Unity Radii**		**VdW Radii** [1–3]		**Covalent Radii** [4]	
**Molecule**	**Charge**	**Std.Dev.**	**Charge**	**Std.Dev.**	**Charge**	**Std.Dev.**
Benzene	0	0.092	0	0.012	0	0.061
Methanol	0	0.081	0	0.015	0	0.049
Phenol	0	0.112	0	0.016	0	0.070
**IL**						
$[EMIm]^+$	0.403	0.109	0.804	0.018	0.583	0.066
$[OAc]^-$	−0.403	0.094	−0.804	0.015	−0.583	0.057
**ILW**						
$[EMIm]^+$	0.515	0.098	0.816	0.020	0.645	0.062
$[OAc]^-$	−0.141	0.110	−0.759	0.017	−0.421	0.064
Water	−0.125	0.081	−0.019	0.015	−0.075	0.047

Table S2: Atomic partial charges and standard deviations for the first three systems from Table 1 in the manuscript, using three different sets of non-optimized standard radii and a one-step Voronoi integration. All numbers in units of e. For atom labels, see Figure 4.

	**Unity Radii**		**VdW Radii** [1–3]		**Covalent Radii** [4]	
**Atom**	**Charge**	**Std.Dev.**	**Charge**	**Std.Dev.**	**Charge**	**Std.Dev.**
**Benzene**						
C	0.501	0.025	−0.757	0.028	0.005	0.014
H	−0.501	0.033	0.757	0.028	−0.005	0.018
**Methanol**						
C	1.519	0.033	−2.467	0.065	0.126	0.019
HC	−0.445	0.022	0.741	0.033	0.002	0.015
O	0.262	0.064	−0.466	0.055	−0.258	0.033
HO	−0.447	0.041	0.710	0.025	0.126	0.016
**Phenol**						
C1	0.238	0.017	−0.354	0.051	0.124	0.012
C2	0.481	0.028	−0.783	0.031	−0.018	0.016
C3	0.511	0.028	−0.750	0.029	0.014	0.015
C4	0.496	0.029	−0.770	0.032	−0.005	0.016
H2	−0.498	0.032	0.763	0.029	0.003	0.018
H3	−0.504	0.035	0.761	0.029	−0.004	0.019
H4	−0.513	0.036	0.760	0.030	−0.010	0.019
O	0.241	0.061	−0.325	0.076	−0.226	0.032
HO	−0.443	0.049	0.709	0.027	0.128	0.023

Table S3: Atomic partial charges and standard deviations for system "IL" from Table 1 in the manuscript, using three different sets of non-optimized standard radii and a one-step Voronoi integration. All numbers in units of e. For atom labels, see Figure 4.

	**Unity Radii**		**VdW Radii**[1–3]		**Covalent Radii**[4]	
**Atom**	**Charge**	**Std.Dev.**	**Charge**	**Std.Dev.**	**Charge**	**Std.Dev.**
**[EMIm]$^+$**						
N1	−0.069	0.017	1.163	0.059	0.096	0.022
C2	0.666	0.030	−1.514	0.048	0.032	0.016
N3	−0.057	0.018	1.177	0.061	0.106	0.023
C4	0.564	0.027	−1.102	0.042	0.001	0.016
C5	0.560	0.028	−1.100	0.042	0.000	0.016
C6	1.069	0.033	−1.654	0.053	0.095	0.018
C7	1.360	0.032	−2.196	0.048	0.018	0.018
C8	1.457	0.036	−2.408	0.059	0.063	0.020
H2	−0.466	0.037	0.805	0.027	0.057	0.016
H4	−0.469	0.033	0.795	0.026	0.047	0.015
H5	−0.480	0.029	0.759	0.031	0.004	0.016
H6	−0.480	0.030	0.760	0.031	0.004	0.015
H7	−0.469	0.028	0.745	0.030	−0.009	0.016
H8	−0.459	0.030	0.763	0.031	0.013	0.016
**[OAc]$^-$**						
C1'	0.487	0.013	−1.118	0.062	0.205	0.013
C2'	1.338	0.033	−2.207	0.053	0.011	0.019
H'	−0.474	0.028	0.736	0.031	−0.017	0.017
O'	−0.403	0.052	0.156	0.068	−0.374	0.033

Table S4: Resulting atomic partial charges and standard deviations for system "ILW" from Table 1 in the manuscript, using three different sets of non-optimized standard radii and a one-step Voronoi integration. All numbers in units of e. For atom labels, see Figure 4.

	**Unity Radii**		**VdW Radii** [1–3]		**Covalent Radii** [4]	
**Atom**	**Charge**	**Std.Dev.**	**Charge**	**Std.Dev.**	**Charge**	**Std.Dev.**
**$[EMIm]^+$**						
N1	−0.068	0.018	1.167	0.065	0.098	0.024
C2	0.676	0.027	−1.517	0.049	0.035	0.016
N3	−0.057	0.019	1.179	0.061	0.107	0.023
C4	0.564	0.026	−1.097	0.043	0.004	0.016
C5	0.566	0.026	−1.100	0.041	0.001	0.015
C6	1.073	0.029	−1.652	0.051	0.098	0.017
C7	1.359	0.030	−2.189	0.043	0.020	0.016
C8	1.460	0.034	−2.397	0.056	0.068	0.019
H2	−0.452	0.032	0.804	0.024	0.064	0.014
H4	−0.458	0.030	0.794	0.024	0.053	0.013
H5	−0.471	0.026	0.758	0.029	0.008	0.014
H6	−0.473	0.026	0.759	0.029	0.008	0.013
H7	−0.466	0.027	0.744	0.029	−0.007	0.015
H8	−0.450	0.026	0.762	0.029	0.017	0.015
**$[OAc]^-$**						
C1'	0.498	0.012	−1.087	0.060	0.219	0.014
C2'	1.342	0.032	−2.193	0.049	0.014	0.017
H'	−0.465	0.026	0.739	0.030	−0.008	0.015
O'	−0.293	0.070	0.152	0.071	−0.315	0.036
**Water**						
OW	0.723	0.067	−1.419	0.036	−0.324	0.033
HW	−0.424	0.042	0.700	0.027	0.125	0.016

References

(1) Bondi, A. van der Waals Volumes and Radii. *J. Phys. Chem.* **1964**, *68*, 441–451.

(2) Rowland, R. S.; Taylor, R. Intermolecular Nonbonded Contact Distances in Organic Crystal Structures: Comparison with Distances Expected from van der Waals Radii. *J. Phys. Chem.* **1996**, *100*, 7384–7391.

(3) Mantina, M.; Chamberlin, A. C.; Valero, R.; Cramer, C. J.; Truhlar, D. G. Consistent van der Waals Radii for the Whole Main Group. *J. Phys. Chem. A* **2009**, *113*, 5806–5812.

(4) Cordero, B.; Gómez, V.; Platero-Prats, A. E.; Revés, M.; Echeverría, J.; Cremades, E.; Barragán, F.; Alvarez, S. Covalent Radii Revisited. *Dalton Trans.* **2008**, *21*, 2832–2838.
